# Computational Studies of Venom Peptides Targeting Potassium Channels

**DOI:** 10.3390/toxins7124877

**Published:** 2015-12-01

**Authors:** Rong Chen, Shin-Ho Chung

**Affiliations:** Research School of Biology, Australian National University, Acton ACT 2601, Australia; shin-ho.chung@anu.edu.au

**Keywords:** potassium channels, peptide toxins, molecular modeling, molecular dynamics

## Abstract

Small peptides isolated from the venom of animals are potential scaffolds for ion channel drug discovery. This review article mainly focuses on the computational studies that have advanced our understanding of how various toxins interfere with the function of K^+^ channels. We introduce the computational tools available for the study of toxin-channel interactions. We then discuss how these computational tools have been fruitfully applied to elucidate the mechanisms of action of a wide range of venom peptides from scorpions, spiders, and sea anemone.

## 1. Overview of Ion Channels and Venom Peptides

Ion channels allow certain ions to move across cell membranes. They are involved in a range of physiological processes such as electrical signaling in muscles and neurons, and their malfunction is implicated in various human diseases. As such, ion channels are one of the most important drug targets [1,2,3]. For example, two isoforms of K^+^ channels, Kv1.3 and K_Ca_3.1, are promising targets for the treatment of autoimmune diseases [4], whereas local anesthetics primarily target Na^+^ channels involved in pain pathways [5].

Animal venoms provide a rich resource from which modulators of a wide array of ion channels can be isolated. Many short peptides from scorpion venoms are potent and specific inhibitors of certain K^+^ channels. For example, charybdotoxin (ChTx) from the deathstalker scorpion is a potent blocker of two isoforms of K^+^ channels, Kv1.3 and K_Ca_3.1, which are potential targets for immunosuppression [6]. The toxin ADWX-1 (Autoimmune Drug from Wen-Xin Group), developed by Li and coworkers [7], has picomolar affinities for Kv1.3 and yet it is more than 300-fold selective for this channel over two highly related channels, Kv1.1 and Kv1.2. Similarly, a family of small peptides isolated from the venom of cone snails, referred to as µ-conotoxins, are potent inhibitors of Na^+^ channels, which are targets for the treatment of pain [8,9]. These venom peptides are promising leads for drug development [10,11,12].

The structure-activity relationship of various toxins, especially the ones that are specific for K^+^ and Na^+^ channels, has been an area of intensive study. Several research teams have focused on the isolation and characterization of toxins from venomous animals, focusing on marine invertebrates [13], arachnids [14,15,16,17,18,19,20], and reptiles [21,22]. Li and coworkers have made many seminal contributions to our understanding of the mechanisms of action of scorpion toxins on voltage-gated K^+^ (Kv) channels [7,23,24,25,26,27,28,29,30,31]. They have isolated and characterized a large number of scorpion toxins and showed how one such toxin could be modified to enhance its potency and selectivity to Kv1.3 [7].

The direct interactions between a toxin and a channel are difficult to probe using available experimental methods but can be examined in atomic detail using computational methods. Therefore, computational tools have been used extensively in recent years to aid the interpretation of experimental data and guide the design of new experiments. Most of the computational studies reported have focused on K^+^ channels whose structures are better understood than other cation channels [32,33,34].

This review article primarily focuses on the computational approaches used to aid our understanding of how various toxins interfere with the function of Kv channels. The paper is organized as follows. We first describe briefly the general architecture of K^+^ channels and introduce computational tools commonly used to study toxin-channel interactions. We then show how these computational tools have been applied to elucidate the mechanisms of action of venom peptides on Kv channels. Finally, we discuss how the principles of toxin-channel interactions learned from those studies may be used for the development of toxin analogs as novel drug scaffolds.

## 2. Structure of K^+^ Channels

In the past two decades, the molecular structures of several isoforms of K^+^ channels, such as the bacterial K^+^ channel KcsA [35], the calcium-gated K^+^ channel MthK [36], the voltage-gated K^+^ channels KvAP [37] and Kv1.2 [38], and the inward-rectifying channel Kir2.2 [39], have been determined from crystallographic analyses. These structures allow the homology modeling of a broad range of K^+^ channels and enable detailed analysis of toxin-channel interactions using computational methods.

**Figure 1:**
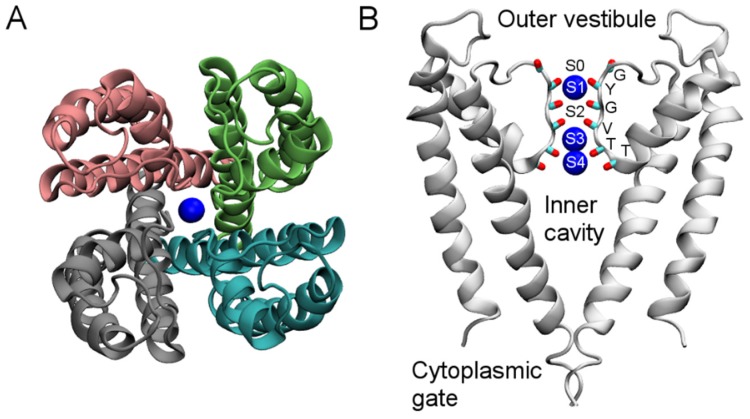

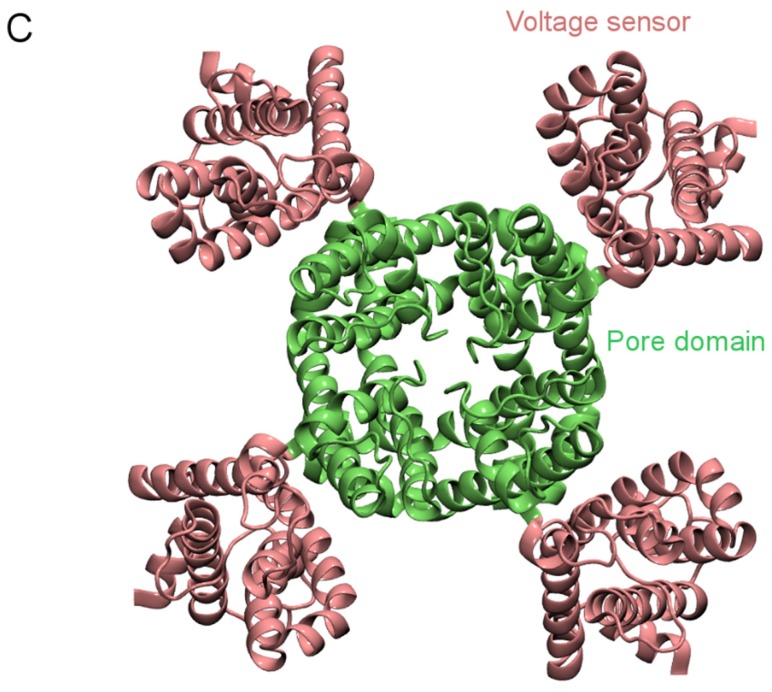
Structure of KcsA (PDB ID 1BL8 [35]) viewed parallel to the channel axis from the periplasmic side (**A**) and perpendicular to the channel axis (**B**). Blue spheres represent K^+^ ions. In (**B**), only two channel subunits are shown for clarity. In (**C**), the crystal structure of the Kv1.2-Kv2.1 paddle chimera channel (PDB ID 2R9R [41]) viewed from the periplasmic side along the channel axis is shown.

KcsA only has a pore domain consisting of four identical subunits (Figure 1A). The channel is composed of three segments. In the middle of the pore domain is the selectivity filter, approximately 12 Å in length and 2.8 Å in diameter. The filter is lined with the backbone carbonyl groups of six residues, TTVGYG, which is a fingerprint sequence and highly conserved across K^+^ channels (Figure 1B). The space between the planes of the six residues forms five K^+^ binding sites, designated as S0–S4. In the crystal structure, the S1, S3, and S4 sites are occupied by K^+^ ions (Figure 1B). Just below the selectivity filter is a water-filled inner cavity of ~5 Å in radius that extends toward the intracellular space (Figure 1B). The pore domain of Kv1.2 is similar to that of KcsA, but each subunit of Kv1.2 also carries a voltage sensor attached to the pore domain (Figure 1C). The voltage sensor moves within the membrane in response to changes in membrane potential, resulting in channel closing or opening. Venom peptides thus can interact with K^+^ channels in two different ways. They either bind to the wall of the external vestibule and the selectivity filter, thereby physically occluding the ionic pathway, or target the voltage sensor, thus interfering with the gating behavior of the channels. Recently, the first crystal structure of a pore-blocking scorpion toxin bound to a Kv channel was solved [40], providing excellent reference for both computational and further experimental studies.

## 3. Computational Methods

Parallel to the advances in understanding channel structures in atomic detail, there have also been important advances in computational biophysics. As new analytical methods have been developed and the available computational power increased, it became possible to unravel the direct interactions between toxins and ion channels using the state-of-the-art computational tools. Interacting residue pairs of a specific toxin and an ion channel can be determined, and their binding affinity can be calculated accurately.

The study of toxin-channel interactions typically consists of two steps. First, the most favorable binding modes between a toxin and a channel are predicted using different methods such as molecular docking [30], Brownian dynamics (BD) [42] and molecular dynamics (MD) [43], often with the help of available experimental data. Then, the dissociation constant (*K*_d_) is calculated as a means to validate the binding modes predicted. In electrophysiological experiments, the half-maximal inhibitory concentration (IC_50_) or the half-maximal effective concentration (EC_50_), instead of *K*_d_, is typically determined. IC_50_ and EC_50_ measure how strongly a toxin modulates the gating of a channel, whereas *K*_d_ reflects how strongly a toxin binds to a channel. Thus, IC_50_ (or EC_50_) and *K*_d_ are different quantities by definition. However, the binding of a toxin to a channel usually implies modulation to the channel activity. Therefore, IC_50_ (or EC_50_) and *K*_d_ are expected to be in the same order in these systems, and have been used interchangeably in literature.

### 3.1. Prediction of Binding Modes

The three most widely used computational methods for the prediction of toxin-channel complex structures, namely, molecular docking, Brownian dynamics (BD), and molecular dynamics (MD), are compared in Table 1. Molecular docking is a computational algorithm that aims to predict complexes between the ligand (toxin) and the receptor (ion channel). Compared with MD, docking methods utilize various ligand sampling techniques such as shape matching, systematic search, and stochastic sampling to enable the rapid prediction of a large number of likely toxin-channel structures with low computational cost. The flexibility of channel and toxin is ignored in rigid-body docking for computational efficiency, although some docking methods allow limited flexibility [44]. The output of docking calculations is a list of docked complexes or poses of the toxin with the channel protein, and the poses are ranked according to an empirical function. For a detailed review of molecular docking algorithms, the reader is referred to Huang *et al.* [45].

**Table 1:** Comparison of three computational methods: docking, Brownian dynamics (BD) and molecular dynamics (MD).

Items	Docking	BD	MD
Water	Implicit	Implicit	Explicit
Ions	Ignored	Explicit	Explicit
Membrane	Ignored	Implicit	Explicit
Flexibility	Ignored/Limited	Ignored	Allowed
Output trajectory	No	Yes	Yes
Time scale	-	Microseconds	Nanoseconds

In BD, two simplifying assumptions are made. First, the water is treated as a continuum. Instead of simulating water molecules explicitly, as in MD, the effects of incessant collisions between ions and water molecules are lumped together, and are represented as frictional and random forces. Second, the internal degrees of freedom in the ion channel and the toxin are ignored. The channel is placed at a fixed position, while the tumbling and translocation but not conformational changes of the toxin are allowed. Therefore, similar to docking methods, the predictions of BD are influenced by the structures of the channel and the toxin used.

In classic MD, the trajectories of N particles interacting via a many-body potential are traced using Newton’s equation of motion. All the molecules in the simulation assembly, including water molecules and ions, are represented explicitly. The interactions between all atoms or particles in the system are described by an empirical potential energy function. This potential function contains harmonic terms for bonds and angles, a cosine expansion for torsion angles, and Lennard-Jones and Coulomb interactions for non-bonded interactions. The potential energy function, together with a set of parameters such as equilibrium bond lengths and angles required for describing interatomic interactions, are referred to as a force field. The accuracy of the force field is essential for a realistic simulation of a biomolecular system. The primary result of MD simulations is a trajectory of all particles in time, from which specific details of the assembly can be analyzed. Thus, the motion of each particle can be followed as it moves in real time on a timescale up to a microsecond, although longer simulations have also been reported.

Once the toxin-channel bound complex is determined, using any one of the three procedures described above, the system is further equilibrated with MD to allow the complex to evolve to a stable state. Atoms can be followed as they move in real time and thus it is possible to identify amino acid side chains on the toxin forming hydrogen bonds with those on the channel, as well as the residue pairs making hydrophobic interactions. This information can be used to validate the binding mode predicted computationally against available experimental data and guide the design of further mutagenesis experiments. Furthermore, the binding affinity (*K*_d_) can be calculated and compared to the IC_50_ or EC_50_ values determined experimentally to further verify the predictions.

Although MD allows the interactions and dynamics in a molecular system to be examined in atomic detail, it has several important limitations. First, it requires sufficient experimental data to be available for constructing the molecular system and validating the computational predictions. However, our knowledge on the structure of ion channels is still limited. For example, no crystal structure for a mammalian Na^+^ and Ca^2+^ channel has been reported. Second, the accuracy of its predictions is limited by the quality of the force field used to describe the interatomic forces in the system. Although the force fields for biomolecular simulations have evolved over the past decade, certain aspects such as the treatment of atomic polarizability are yet to be implemented in any of the most widely used biomolecular force fields such as CHARMM and GROMOS. Third, the time scale of the MD simulation that can be achieved is limited by the computational resources available. For a comprehensive discussion on the limitations of MD, see van Gunsteren *et al.* [46].

### 3.2. Calculation of K_d_

One of the major aims of computational studies is to calculate *K*_d_ accurately, because it is not only a central quantity for describing toxin block, but also the only quantity that is directly accessible to available experimental techniques. The potential of mean force (PMF) profile of toxin binding to a channel, which provides the work required to move a toxin along the channel axis, can be used to derive the *K*_d_ value of toxin binding. Typically, the well in the PMF profile points to the binding site in the channel and the depth of the profile largely determine the *K*_d_. The formal link between the PMF profile and *K*_d_ has been derived using different statistical mechanical methods [32,47]. The *K*_d_ can be calculated from the PMF, *W(z)*, using the following equation:

(1) $$K_{\text{d}}^{-1}=1000\pi R^{2}N_{\text{A}}\int_{z_{\min}}^{z_{\max}}\exp[-W(z)/kT]\text{d}z$$

where π*R^2^* is the cross-sectional area sampled by the center of mass (COM) of the toxin, *N*_A_ is Avogadro’s number, *z_min_* and *z_max_* are the *z* positions of the toxin COM when the toxin is fully bound to the channel and in the bulk, respectively.

The PMF can be calculated most simply from the definition of work as a line integral of force. In the simulation the COM of the toxin is constrained with a spring constant to a reference point that moves slowly with a constant velocity. The work performed by the spring as a function of the toxin position is obtained. The simulation is then repeated many times and the ensemble average of the work is used to construct the PMF profile according to Jarzynski’s equality [48]. The reference point must move slow enough such that the work performed is reversible and can be used to estimate the PMF accurately. If the reference point moves too fast, the work would be too large and not measure the PMF correctly [49].

In order to construct the PMF profile efficiently, the umbrella sampling technique is widely used. From the bound position to the channel, the toxin is slowly pulled out along the channel axis. A series of structures with the COM of the toxin spaced at small intervals (typically 0.5 Å) is generated. Each of these structures is viewed as an umbrella window and simulated independently. In each umbrella window the COM of the toxin is harmonically restrained to the starting position, so that the sampling of toxin COM is focused on a narrow region. The COM of the toxin is also restrained to a small cylinder (typically 8 Å in radius) centered on the channel axis using a flat-bottom harmonic potential. The cylinder must be wide enough such that the restraining potential is always zero when the toxin is bound to the channel and does not interfere with sampling. The distribution of toxin COM can be derived from each simulation and then unbiased to obtain the correct PMF profile [50].

At the fully bound position, the degrees of freedom of the toxin are restricted by the toxin-channel interactions. Therefore, the PMF of the toxin would largely be determined by the interactions in the complex predicted. If the complex predicted is the most favorable one, the *K*_d_ value predicted from the PMF profile should be in close proximity to the IC_50_ value determined experimentally. On the other hand, if the complex is not favorable, the experimental IC_50_ value would not be reproduced. Thus, calculations of IC_50_ can help verify the binding mode predicted.

## 4. Venom Peptides

### 4.1. Scorpion Toxins

Many small peptides of 30–70 residues from scorpion venoms have been isolated and characterized. These peptides can be broadly classified into two classes: pore blockers of K^+^ channels and gating modifiers of Na^+^ channels. The pore blocking peptides are typically 30–40 residues in length. They bind to the outer vestibule and physically occlude the selectivity filter of the channel, thereby inhibiting ion permeation. On the other hand, scorpion α- and β-toxins, which are well characterized gating modifiers of Na^+^ channels, typically comprise of 60–70 residues. Scorpion α- and β-toxins bind to the voltage sensor of the channel and modulate the movement of the voltage sensor in response to changes in membrane potential.

**Figure 2:**
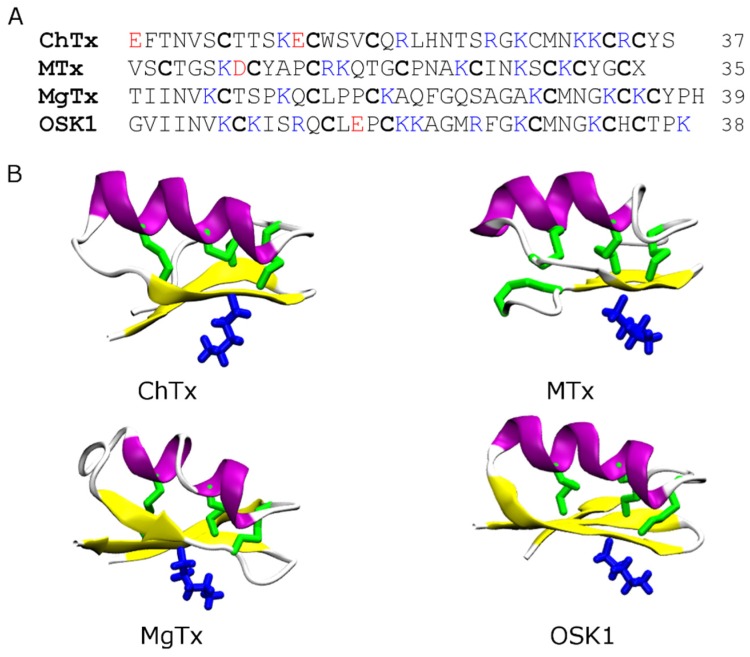
Primary (**A**) and secondary (**B**) structures of four scorpion toxins: charybdotoxin (ChTx, PDB ID 2CRD [56]), maurotoxin (MTx, PDB ID 1TXM [57]), margatoxin (MgTx, PDB ID 1MTX [58]) and OSK1 (PDB ID 1SCO [59]). The disulfide bridges are shown in green and the key pore-blocking lysine residue in blue. The α-helix is shown in purple and β-strand in yellow.

The majority of the scorpion toxins that have been tested on ion channels belong to the pore blockers category. Among the most well studied are charybdotoxin (ChTx) [51], maurotoxin (MTx) [52], margatoxin (MgTx) [53], OSK1 [54], and ADWX-1 [7]. All these toxins are potent blockers of Kv1.3, a target for autoimmune diseases [6]. They share a common structural scaffold consisting of an α-helix and two or three anti-parallel β-strands (Figure 2). They all carry six or eight cysteine residues forming three or four disulfide bridges, and multiple lysine and arginine residues that are positively charged at neutral pH. However, several scorpion toxins of different structural folds have also been shown to have inhibitory effects on K^+^ channels [25,55]. For example, Li and coworkers discovered that the scorpion toxin Hg1 [25], carrying two anti-parallel β-strands and one α-helix consistent with the Kunitz type fold, is a potent inhibitor of Kv1.3 (*K*_d_ = 6 nM).

**Figure 3:**
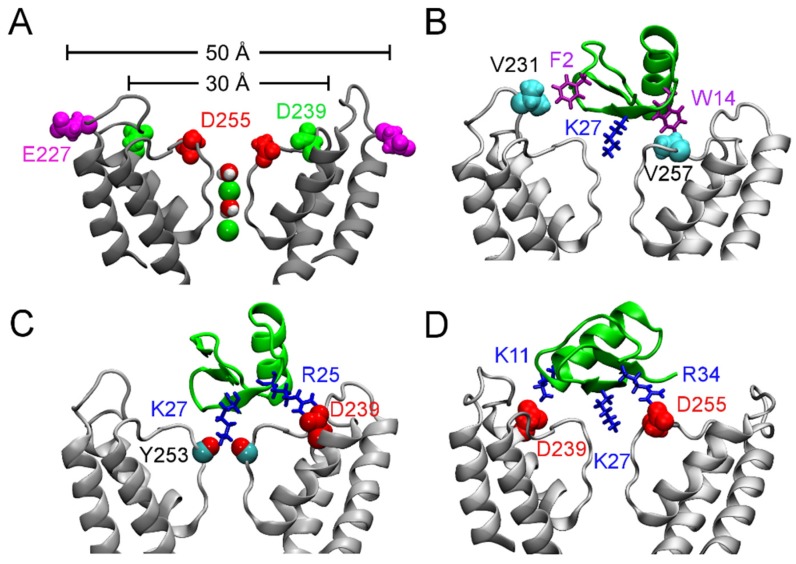
Binding of ChTx to the Ca^2+^-activated K^+^ channel of intermediate conductance, K_Ca_3.1. (**A**) Structure of K_Ca_3.1 modeled on Kv1.2 viewed perpendicular to the channel axis. Only two of the four subunits are shown for clarity. The position of three acidic residues relevant for scorpion toxin binding are highlighted. (**B**–**D**) ChTx (green) bound to K_Ca_3.1, with the most important contacts highlighted. Adapted from Chen *et al.* [70], Copyright 2013, Elsevier.

The pore-blocking mechanisms of scorpion toxins for K^+^ channels have been examined extensively using computational methods [29,30,42,43,60,61,62,63,64,65,66,67,68,69,70]. In these studies different methods such as molecular docking [30,66,69], Brownian dynamics [42,60], and MD simulation with distance restraints [43,67,68,70] were used to predict the binding modes between the toxin and the channel. Although the methods are of a different level of complexity and accuracy, the general principles for toxin-channel association predicted from these methods were consistent. In general, the key lysine residue from the middle of the β-strand of the toxin, which is preceded by the α-helix (Figure 2), protrudes into the selectivity filter of the channel, thereby physically occluding the permeation pathway. Additional hydrophobic and electrostatic contacts are also formed to stabilize the toxin-channel complex (Figure 3).

**Figure 4:**
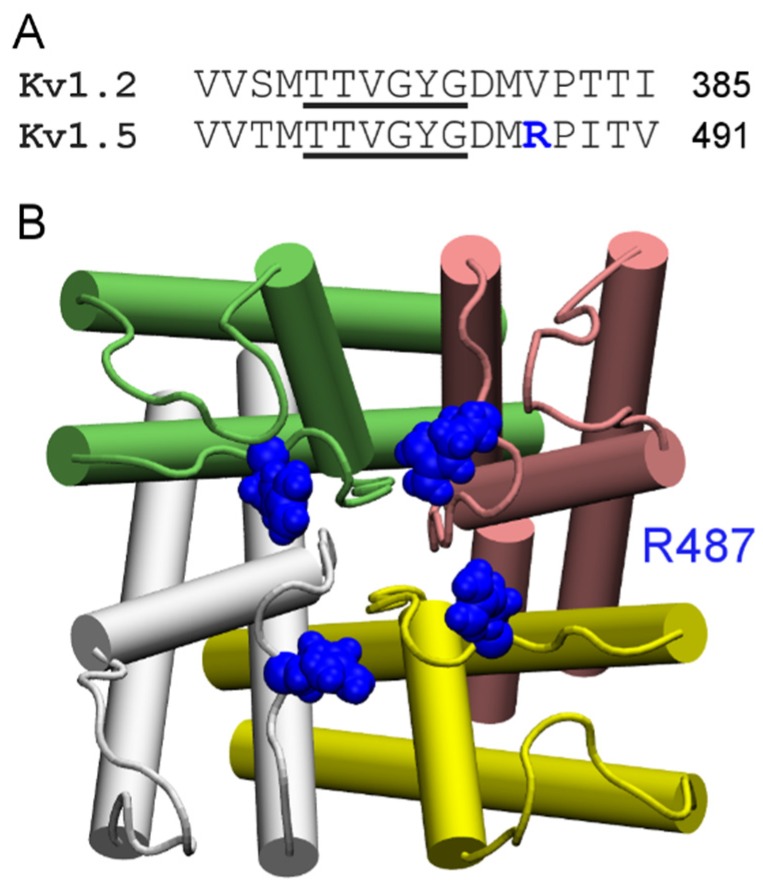
Sequence and structure of the ChTx-insensitive channel Kv1.5. (**A**) Sequence alignment of human Kv1.2 and human Kv1.5 in the filter (underscored) region; (**B**) Structure of Kv1.5 modeled on the crystal structure of Kv1.2 (PDB ID 3LUT [71]) viewed from the periplasmic side along the channel axis. The Arg487 residue just outside the filter is highlighted. The four channel subunits are shown in the Cartoon representation and different colors.

According to their sensitivity to the classic scorpion toxin ChTx, K^+^ channels can be classified into two groups. The first group comprises of K^+^ channels such as Kv1.1, Kv1.2, Kv1.3, and K_Ca_3.1 that are extremely sensitive to ChTx, with IC_50_ values in the nanomolar range. In contrast, KCNQ1, Kv1.5, and Kv4.3, which are channels of the second group, are insensitive to ChTx. Channels of the first group generally carry several rings of acidic residues in the outer vestibule which interact favorably with the highly basic toxins. For example, the outer wall of K_Ca_3.1 carries three rings of acidic residues (Figure 3A). Once ChTx is bound, in addition to hydrophobic interactions (Figure 3B), several hydrogen bonds are formed between the acidic residue of the channel and basic residues of the toxin (Figure 3C,D). These hydrogen bonds play crucial role in stabilizing the complex. However, Kv1.5 carries a ring of arginine residues just outside the filter, at the position corresponding to V381 of Kv1.2 (Figure 4). The positive charges carried by the arginines could render the channel insensitive to ChTx, which carries a net charge of +5 *e*. Thus, toxins active on channels of the second group must carry acidic residues. Consistent with this prediction, several toxins with micromolar affinities for KCNQ1 discovered by Li *et al.* [26] all carry multiple acidic residues.

### 4.2. Conotoxins

In over 50 million years of evolution, marine cone snails have developed a vast array of toxins for prey capture and defense. Only a small fraction of conotoxins has been characterized so far. There are over 500 species of cone snails, each species carrying over 100 different conotoxins [72]. A major component of the cone snail venom is peptide toxins. Based on primary structure, these toxins have been classified into seven superfamilies, each of which comprise of several subfamilies acting on different types of ion channels [72]. For example, µ-conotoxins belonging to the M superfamily are potent blockers of Na^+^ channels [73], whereas ω-conotoxins of the O superfamily are Ca^2+^ channel blockers [74]. Although the majority of conotoxins that have been characterized are inhibitors of Na^+^ channels, Ca^2+^ channels and nicotinic acetylcholine receptors, several conotoxins, such as ViTx [75] and PVIIA [76], have been shown to block Kv channel currents. The mechanism of action by ViTx is unclear, but PVIIA likely acts on the channel pore directly [77].

**Figure 5:**
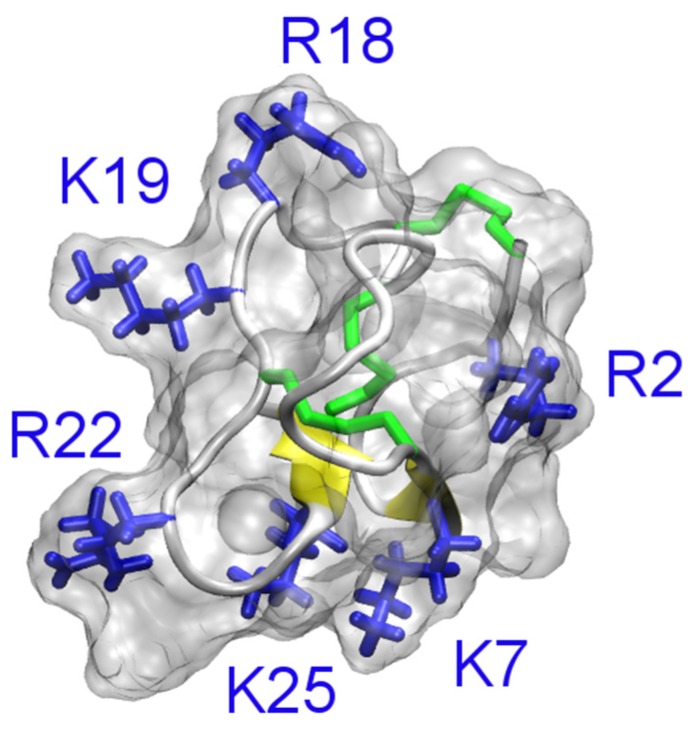
Solution structure of PVIIA (PDB ID 1KCP [77]). The three disulfide bonds are shown in green and β-strands in yellow.

The primary structure of PVIIA is CRIPN-QKCFQ-HLDDC-CSRKC-NRFNK-CV and the C-terminus of the toxin is amidated. The toxin carries six lysine and arginine residues, and thus is positively charged at neutral pH. The tertiary structure of PVIIA is given in Figure 5. It is evident that PVIIA does not carry the α-helix in the N-terminus typical to ChTx-like scorpion toxins. Despite differences in structural folds, PVIIA and scorpion toxins are believed to block K^+^ channels in a similar manner. The binding modes of PVIIIA to the *Shaker* K^+^ channel have been examined in detail using docking and MD by Mahdavi and Kuyucak [78]. The lysine residue at position 7 of PVIIA was found to protrude into the channel filter and physically occlude the permeation pathway, in a way similar to that of scorpion toxins [78,79].

### 4.3. Spider Toxins

Spider venoms are also a rich resource for the isolation of small peptides as potential drug scaffolds [10]. It has been estimated that spider venoms as a whole can provide more than 16 million unique peptides, with each venom of approximately 80,000 species containing 200 unique peptides [80]. However, only a small number of those peptides have been characterized.

**Figure 6:**
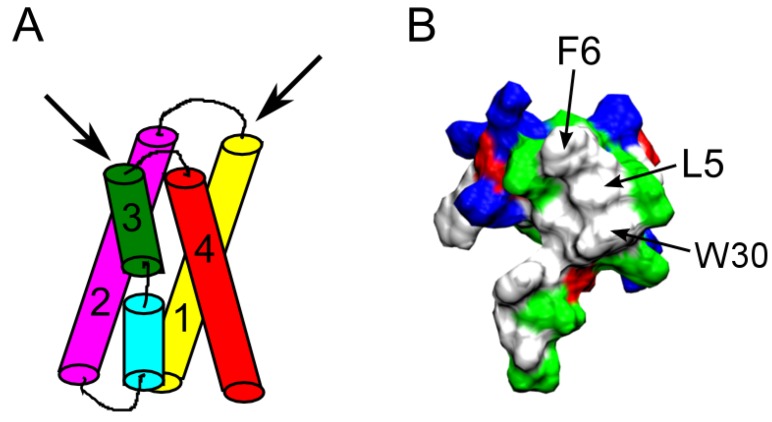
(**A**) Schematic of the voltage sensor domain of voltage-gated K^+^ channels. The four helices forming the voltage sensor, S1-S4, are indicated. The two black arrows denote the two possible directions a gating-modifier toxin may bind to the crevice between the S1-S2 and S3-S4 linking loops at the extracellular end of the voltage sensor. **(B**) Molecular surface of HaTx1 (PDB ID 1D1H [88]) showing three key hydrophobic residues.

Some of the most well characterized spider toxins are Hanatoxin (HaTx) [81], *Scodra griseipes* toxin (SGTx1) [82], heteropodatoxin (HpTx) [83], phrixotoxin (PaTx) [84], and the voltage sensor toxin (VSTx) [85]. All these toxins target the voltage sensor domain, formed by four helices S1–S4 (Figure 6A), within the membrane [86]. This is different from ChTx-like toxins that bind to the pore domain. As such, these toxins are referred to as gating-modifiers. The backbones of these toxins contain three disulfide bonds forming the inhibitor cysteine knot motif, which is also common to ChTx and other pore-blockers [87]. A hydrophobic face primarily formed by phenylalanine, leucine, methionine, and tryptophan residues is conserved across these gating-modifiers (Figure 6B) [88,89,90,91]. This hydrophobic face is believed to be critical for toxin function [86,92,93].

The detailed process by which one of the gating-modifier toxins penetrates into the membrane has been examined in detail using MD simulations [94,95,96,97]. These simulations suggested that the toxins bind preferentially to head group region of the bilayer, consistent with recent experiments [98]. Once bound, the hydrophobic face of the toxin would interact favorably with the hydrophobic core of the bilayer, and the polar region of the toxin with lipid head groups and water molecules. However, the interaction between the toxin and the voltage sensor domain within the membrane is less well studied. A toxin may bind to the extracellular end of the voltage sensor from the side of S2 and S3 helices, or S1 and S4 helices (Figure 6A). MD simulations suggested that HaTx1 may bind to the voltage sensor of Kv2.1 from the S2 and S3 helices [99]. This proposal is in accord with the recent study by Gupta *et al.* [91]. Using the ROSETTA program [100], they constructed the complex structure of the spider toxin Guangxitoxin and the Kv1.2/2.1 paddle chimera channel, and found that this toxin also binds primarily to the S2 and S3 helices [91].

A globular polypeptide toxin, known as GsMTx4, extracted from the tarantula *Grammostola spatulata*, influences the gating kinetics of gramicidin [101] and mechanosensitive channels [102,103]. The mechanism by which this toxin interferes with the gating behavior of the two cationic channels is different from that of other spider toxins we discussed above. Instead of binding to the channel protein, GsMTx4 partitions into the membrane and interfere with the gating mechanisms indirectly through changes in membrane properties. MD simulations suggest that GsMTx4 binds to the interfacial region of the membrane and induces membrane thinning [104]. Notably, experimental evidence indicates that VSTx1 also causes membrane thinning when bound to the headgroup region of the lipid membrane [98]. 

### 4.4. Other Animal Toxins

Several sea anemone toxins such as BgK [105,106] and ShK [107] have been shown to be potent inhibitors of K^+^ channels. BgK blocks Kv1.1, Kv1.2, and Kv1.3 effectively at nanomolar concentrations [108]. ShK is an extremely potent blocker of Kv1.3, with an IC_50_ value in the picomolar range [107]. A domain of the matrix metalloprotease 23 protein from mammals, MMP23_TxD_, shows high sequence similarity with BgK and ShK. However, MMP23_TxD_ from rat is only a weak inhibitor of Kv1.3 (IC_50_ = 2.8 µM) [109].

**Figure 7:**
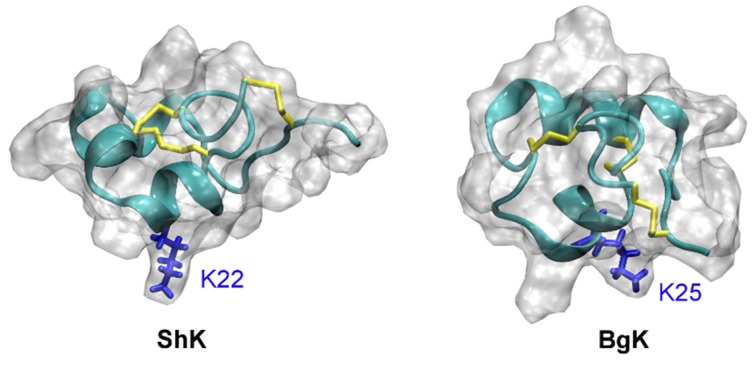
Solution structures of ShK (PDB ID 1ROO [110]) and BgK (PDB ID 1BGK [113]). The three disulfide bonds in each toxin are shown in yellow. The side chain of the key lysine residue is highlighted.

The solution structures of ShK (PDB ID 1ROO [110]) and BgK (PDB ID 1BGK [106]) are shown in Figure 7. The position of the pore-blocking lysine residue for K^+^ channels (Lys22 for ShK and Lys25 for BgK), as inferred from experimental data [111,112], is also indicated in the figure. It is evident that ShK and BgK share a common structural fold, which is significantly different from that of the scorpion toxin ChTx. In these two toxins, two α-helices are connected with three disulfide bonds but no β-strand structure is evident.

ShK has been studied extensively in an attempt to develop novel drugs for the treatment of autoimmune diseases, because it blocks Kv1.3 extremely potently [114,115,116,117]. Several derivatives of ShK, such as ShK-Dap^22^, ShK-186 and ShK192, highly specific for Kv1.3 over other Kv channel isoforms have been reported [114]. For example, ShK-192 is about 1,000-fold selective for Kv1.3 over Kv1.1, while the wild type toxin only has three-fold selectivity. 

Several computational studies are consistent with the Lys22 residue of ShK being the pore-blocking residue for Kv1.3 [64,69]. However, whether Arg11 of the toxin interacts with the aspartate just outside the filter (Asp449) or on the outer vestibule about 10 Å from the filter (Asp433) is unclear [64,69]. Since mutagenesis experiments do not probe the interacting residue pairs in the toxin-channel complex directly, it is difficult to conclude which one of the models is more correct. It should be noted that binding of biomolecules is a dynamic process and different microstates likely co-exist at equilibrium. It has been shown that different binding modes are possible for hongotoxin and Kv1.3 when the same pore-blocking lysine (Lys28) occludes the entrance of the filter [68]. Therefore, it remains to be determined whether multiple binding modes would be required to describe the binding of ShK to Kv1.3.

Recently, several computational studies have focused on tertiapin (TPN), which is isolated from honey bee venoms, and inward-rectifying K^+^ channels [118,119,120,121]. In all these studies, a rigid-body docking method was used for the prediction of toxin-channel complex structures. However, results from rigid-body docking methods are sensitive to the initial structures used. Therefore, inconsistent models of toxin-channel complexes have been proposed in these studies. For example, the 12th conformer of the 21 solution structures of TPN was proposed to dock rKir1.1 most favorably [121]. The Lys17 residue of TPN was predicted to occlude the filter of Kir3.2 [119], whereas different residues (His12 and Lys21) were proposed to be the pore-blocking residue for Kir1.1 [118,120,121]. Whether TPN can block Kir3.2 or Kir1.1 by inserting alternative residues into the channel pore remains to be elucidated. Such a multiple-binding-mode mechanism has been demonstrated for µ-conotoxins and Na^+^ channels [122], but not yet for K^+^ channels.

## 5. Toxin Analogues

Wild type toxins usually lack specificity and act on a range of targets, as specificity is not required for venomous animals to paralyze prey. On the other hand, high specificity is desired for the toxins to be used as drug candidates. Therefore, toxin analogs selectively acting on the drug target must be designed. At least two approaches for developing more specific toxin analogs are possible. Point mutations can be introduced to the toxins that are potent but nonspecific, or molecules that broadly mimick the action of venom peptides on ion channels can be designed *de novo*.

Several mutant toxins with high specificity for Kv1.3 have been successfully developed. For example, OSK1, a peptide of 38 residues isolated from the venom of *Orthochirun scrobiculosus*, is one of the most potent toxins for Kv1.3 (IC_50_ = 14 pM), but it inhibits Kv1.1 equally well (IC_50_ = 14 pM) [54]. The K16D20 double mutant of OSK1 is more than 100-fold selective for Kv1.3 (IC_50_ = 3 pM) over Kv1.1 (IC_50_ = 400 pM) and Kv1.2 (IC_50_ = 3 nM) [54]. A synthetic analog of ShK, ShK(L5), is also more than 100-fold selective for Kv1.3 (IC_50_ = 0.07 nM) over other Kv1.x channels [123]. ShK-Dap22, in which the Lys22 residue of ShK is replaced with a diaminopropionic acid, is about 80-fold selective for Kv1.3 (IC_50_ = 23 pM) over Kv1.1 (IC_50_ = 1.8 nM) [116]. A triple-point mutation of a kaliotoxin enhances the toxin’s specificity for Kv1.3 over Kv1.1 to over 300-fold [31]. Rashid *et al.* computationally designed a mutant HsTx1, HsTx1-[R14A], which was shown experimentally to be more than 2000-fold selective for Kv1.3 over Kv1.1 [124]. Thus it is possible to design highly selective toxin derivatives. The selectivity of a toxin may also be tuned by repositioning its acidic residues, according to the recent results by Wu and coworkers [125].

## 6. Concluding Remarks

The mechanisms of action of a variety of venom peptides on ion channels have been elucidated with the help of experimental and computational methods. Using the available computational tools, it is possible to predict the modes of binding of a toxin to a channel, and compute the binding affinity (*K*_d_) accurately. For example, the *K*_d_ value for the binding of µ-conotoxin PIIIA to the bacterial sodium channel Na_V_Ab was computed to be 20–30 pM [122], within six-fold to the value of 5 pM determined in subsequent experiments [126]. With the advance in the understanding of toxin action on a molecular level, rational design of highly specific toxin analogs will become possible. It is expected that computational methods will continue to play an important role in the development of ion channel drugs from venom peptides.
